# Factors associated with time until metastatic spread in patients with primary uveal melanoma: A retrospective analysis

**DOI:** 10.1371/journal.pone.0328360

**Published:** 2025-08-19

**Authors:** Reinhard Told, Adrian Reumueller, Judith Kreminger, Stefan Sacu, Roman Dunavoelgyi

**Affiliations:** Department of Ophthalmology and Optometry, Medical University of Vienna, Vienna, Austria; Tehran University of Medical Sciences, IRAN

## Abstract

**Purpose:**

To characterize patients with choroidal or ciliary body (CB) melanoma in order to identify factors associated with time until metastasis and further perform a detailed characterization of the factors identified.

**Methods:**

A mixed model approach was used to identify parameters and patient characteristics associated with time until metastatic event. Consequently, the identified parameters were characterized.

**Results:**

In 383 patients with primary choroidal and CB melanoma, tumor thickness and patient age at diagnosis are associated with time until metastatic event. Mean patient age was 61 ± 13 years, mean choroidal and CB melanoma thickness was 5.1 ± 1.9 mm. Other factors investigated comprising sex, primary tumor width, length, volume, shape, and type of therapy are not associated with time until metastatic event in this patient collective.

**Conclusion:**

In conclusion, this study expands the current knowledge on factors associated with metastasis due to primary choroidal or CB melanoma. So far primary choroidal or CB melanoma thickness has been shown to be a risk indicator for metastatic events. Now, we could show that primary choroidal or CB melanoma thickness is also correlated with time until metastatic event. The association can be expressed as: the thicker the primary tumor, the earlier the potential metastatic event. Age may be considered an indirect effect in the model calculated.

## Background

Choroidal melanoma is a relatively rare tumor that arises from melanocytes [1] of the choroid in about 90% of cases, the ciliary body in 7%, and the iris in approximately 3% of patients [2,3]. Nonetheless, it stands as the most common primary intraocular cancer in adults. The patients’ demographic profile, sites of origin [4], clinical course, indicators for and sites of metastatic spread [5], therapeutic outcome as well as survival rates [6] have been well described previously. In short, the annual prevalence rates range from two to eight per million in southern and northern Europe, respectively [7]. The majority of affected individuals are white (94–98%), with around 5% identifying as Hispanic and 1% as Asian [8,9].

Factors that increase the risk for choroidal melanoma include having fair skin that does not tan, as well as possessing lighter eye colors [10,11]. There is substantial documentation regarding the links between choroidal melanoma and conditions such as oculodermal melanocytosis, choroidal nevus, and various genetic risk factors [1,12,13].

In 2–4% of patients, metastases are identified at the time of diagnosis [14,15]. The liver is the most frequently affected site for metastasis, occurring in 91% of cases as a result of hematogenous dissemination, followed by the lungs, bones, and skin [16]. Despite undergoing treatment, around 30% of patients will develop metastases within a span of 10 years [17]. Studies have shown that several factors are important for primary UM metastasis risk. These factors comprise primary tumor thickness, location, size, extraocular extension as well as histologic and cytogenetic factors [5]. In particular primary tumor thickness [18,19] and volume [20] have been identified as key factors predictive of UM related metastasis. This association has later been quantified in more detail as the studies show an increasing risk with each millimeter increase in thickness [5,21]. Additional factors associated with the likelihood of metastatic spread have been reported. First, cytogenetic alterations noted are monosomy 3, along with gains of chromosome 8q and 6q, and losses of 6q, 16q, and 1q [22]. Second, mutations in GNA11, GNAQ, CYSTLTR2 and PLCB4 genes, as well as genetic alterations in additional genes BAP1, SRSF2, SF3B1 or EIF1AX may affect the likelihood of metastatic spread [23,24].

Although, there are several treatment options available [25], there is general consensus, that due to the lethal potential of the disease, treatment should be initiated early and swiftly. Even after treatment, a continuous and lifelong observation is advised [26], to detect changes of the primary tumor, side effects of treatment and potential metastatic spread early.

In this study we conduct a retrospective analysis in 383 patients with primary choroidal or ciliary body (CB) melanoma to identify factors associated with time until metastasis and further perform a detailed characterization of the factors identified.

On the long term, this study has the potential to identify factors that could lay the groundwork for personalized follow-up and treatment plans. Additionally, factors that enhance effective communication with patients may offer short-term benefits.

## Methods

A retrospective review of medical records was conducted on consecutive patients with the clinical diagnosis of choroidal and CB melanoma treated at the Medical University of Vienna between January 1997 and December 2021. Approval of the local ethics committee (EK: 1339/2023, ClinicalTrials.gov: NCT05733728) was obtained and the study adhered to the tenets of the Declaration of Helsinki including current revisions and the Good Clinical Practice (GCP) guidelines. Data were accessed between 10^th^ of January 2024 and May 2024. All data were anonymized by assigning a patient ID number.

Patients were examined by experienced ocular oncologists at each visit using slit-lamp biomicroscopy, indirect ophthalmoscopy, and multimodal imaging. Diagnosis of UM was based on clinical features and ophthalmic imaging. Imaging included wide-angle fundus photography, ultrasonography, fundus autofluorescence, optical coherence tomography (OCT), was well as fluorescein angiography, indocyanine green angiography, and OCT angiography, when necessary.

Demographic data included age (years) and sex (male, female). Data collected included clinical features of the primary tumor and treatment method. Clinical features included location of tumor epicenter (choroid, CB), tumor thickness in millimeters (mm), width (mm, tangential), length (mm, longitudinal), volume (mm^3^ based on the ellipsoidal solid model (π/6 × length × width × height) [27]), primary tumor configuration (dome, mushroom shape). Data describing tumor dimensions were extracted form ultrasound B-echography. Ultrasonography measurements comprise one transverse still-frame trough the apex of the lesion and one still-frame longitudinal image through the greatest expansion of the lesion using a 20 mhz probe for intraocular and orbital imaging. Measurements were acquired with the systems caliper function. The diameters measured on transverse and longitudinal scans were defined as a straight line from one tumor border to the other. Tumor thickness was assessed from the apical tumor surface to the inner sclera. Additionally, time until therapy (weeks) was collected. Treatment methods included plaque radiotherapy using radioactive isotopes made of Ruthenium, enucleation, and stereotactic radiotherapy (LINAC) with five fractions between 5–7 days. Patients received 10 Gy per fraction prescribed to the 80% isodose [28–30]. Choice of treatment was dependent on tumor location, size, clinical features, and patient’s desire.

Follow-up was performed at months three, six, nine and twelve during the first year, and every six months thereafter; more often if needed. Patients underwent screening for metastatic spread in an outpatient setting every six months including a detailed physical examination, blood samples, as well as abdominal ultrasound or computer tomography/magnet resonance tomography or positron emission tomography scan as needed. Patients were released to an outpatient setting 2–5 years after treatment if no change occurred. Available reports from the outpatient follow-ups every 6 months were used together with in-house data in the follow-up analysis. Patients whose medical records lacked data or were lost to follow-up were not included in this study, or were excluded from the follow-up analysis.

Statistical analysis was performed using SPSS Statistics (Version 26.0. Armonk, NY: IBM Corp.). Descriptive statistics are reported as the mean ± standard deviation (SD).

**Fig 1:**
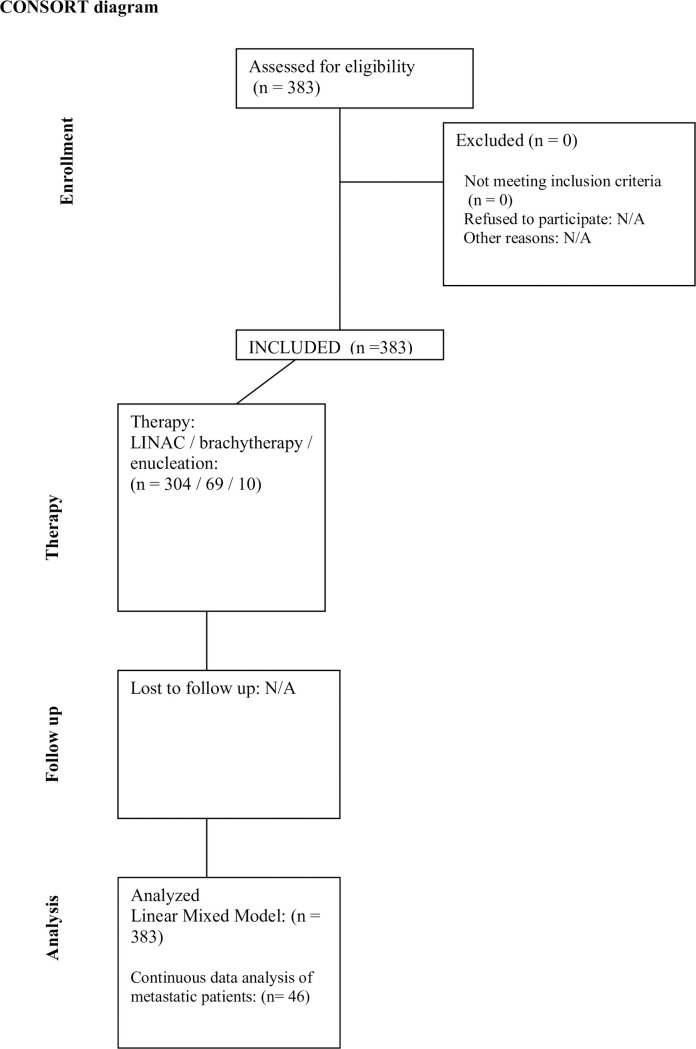
CONSORT diagram.

Evaluation for normal distribution was done using box plots and the Shapiro-Wilk test. We calculated a linear mixed model including a random effect for the patient, to assess the associations between time until metastatic spread and demographic data, primary tumor clinical features and treatment methods. A random intercept for each subject was used to address correlated data. A repeated measures ANOVA with Bonferroni correction was used to assess change over time. T-test and one way-ANOVA were used to compare means. Pearson and Spearman correlation were used determine associations between variables. The level of significance was set to α = 0.05. Fig 1 shows the CONSORT diagram.

## Results

We identified 383 patients treated with LINAC-based hypofractionated stereotactic photon radiotherapy, brachytherapy (Ruthenium-125) or enucleation due to primary choroidal or CB melanoma. 71 (18.5%) of these patients reached documented metastatic disease. Table 1 shows baseline characteristics of patients with primary choroidal or CB melanoma. Table 2 shows baseline T-category AJCC 8th edition staging.

**Table 1:** Characteristics of patients with primary choroidal or CB melanoma; Data are displayed as mean ± SD, n = number of patients.

Patient demographics	
n	383
Age (years)	61 ± 13
Sex (f / m)	186 / 197 (51 / 49%)
**Primary tumor characteristics**	
Choroidal / ciliary body melanoma	357 / 26
Thickness (mm)	5.1 ± 1.9
Width (mm)	10.7 ± 5.8
Length (mm)	11.9 ± 2.7
Volume (mm^3^)	377.7 ± 271.8
Dome- / mushroom shape	330 / 53
LINAC / brachytherapy / primary enucleation	304 / 69 / 10
Time until therapy (weeks)	5.7 ± 12.8

**Table 2:** Primary tumor T-category (thickness) based on AJCC 8th edition staging system [31]: ‘a’ represents choroidal melanoma, ‘b’ represents CB melanoma. Categories 1 to 4 are based on primary tumor thickness and largest basal diameter. Total percent sum exceeds 100% due to rounding error. Primary analysis contains all 383 patients, while secondary analysis focusses on continuous data of patients with metastatic disease. Out of 71 patients with metastatic disease, continuous data was available from 46 patients.

	Number (n)	Percent (%)		
	Primary analysis		Secondary analysis	
	n = 383	%	n = 46	%
T1a	74	19.3	8	17.4
T2a	225	58.9	26	56.5
T3a	55	14.3	11	23.9
T4a	3	0.8	0	0
T1b	3	0.8	0	0
T2b	9	2.5	0	0
T3b	14	3.8	1	2.2
T4b	0	0	0	0

We first used a linear mixed model to identify parameters associated with the time to a metastatic event in choroidal or ciliary body (CB) melanoma. The linear mixed model shows that primary choroidal and CB melanoma thickness (p = 0.014, estimation of fixed effects CI: 0.09 - 0.73), as well as patient age (p = 0.006, estimation of fixed effects CI: −1,25 - −0.13) were statistically significantly associated with the time until a metastatic event (ME). Variables primary tumor width (p = 0.53), length (p = 0.91), volume (p = 0.82), shape (dome, mushroom shape; p = 0.87), as well as patient sex (p = 0.802), each were not statistically significantly associated with the time until a MEfollowing primary choroidal or CB melanoma. Time until therapy (weeks) as well as treatment type were added as confounders to the model. Time until therapy increases the significance as compared to calculating the model without the confounder; UV melanoma thickness (p = 0.044) and patient age (p = 0.03) remain statistically significant. Treatment type has no influence on the results of the analysis.

**Fig 2:**
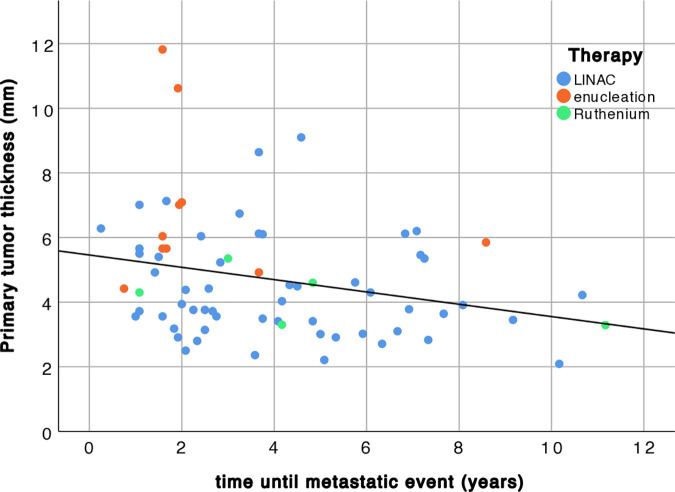
Correlation between primary tumor thickness (mm) and time until metastatic event (years) in 71 patients.

71 of 383 patients (18.5%) had documented metastatic disease following primary choroidal or CB melanoma. Metastatic spread occurred after a mean of 4.8 ± 3.5 years. The sites of metastasis were liver (n = 62; 87%), bones (n = 3; 4%), lung (n = 2; 3%) and multiple sites of metastasis (n = 4; 6%). There is no statistically significant difference in time until ME between therapies used (LINAC, Ruthenium, enucleation) in a one-way ANOVA (p = 0.18, see Fig 2 for therapy in relation to primary tumor thickness).

In a second step, our analysis focused on characterizing previously identified parameters: patient age and primary tumor thickness.

### Age

Comparing age at choroidal or CB melanoma diagnosis between patients with (60.5 ± 14.6 years) and without ME (61.6 ± 13.1 years) shows, that there is no statistically significant difference between groups (p = 0.54). There is no correlation of age at diagnosis with T-staging (Table 2) in both groups, metastatic (Spearman r = −0.03, p = 0.87) and non-metastatic (r = −0.12, p = 0.1). Consequently, this finding is mirrored when correlating primary tumor thickness and age; metastatic group Pearson r = −0.07, p = 0.58 and non-metastatic group Pearson r = −0.07, p = 0.28.

### Thickness

Comparing primary choroidal or CB melanoma thickness between patients with (4.7 ± 1.8 mm) and without ME (5.1 ± 1.8 mm) at the time of diagnosis shows, that there is no statistically significant difference between groups (p = 0.061). There is a statistically significant (p = 0.03) and negative (r = −0.27) correlation between primary tumor thickness and time until metastasis (see Fig 2). The mean time until metastasis is 3.2 ± 2.3 years in patients with a primary choroidal or CB melanoma thicker than the cohort mean (4.7 ± 1.2 mm), whereas patients with a primary tumor thinner than the cohort mean experience a ME after a mean of 4.5 ± 2.7 years. The difference between means is statistically significant (p = 0.045).

Out of 71 patients with metastatic disease, continuous data was available from 46 patients. Of these 46 patients, 45 (98%) presented with primary choroidal melanoma and one (2%) with a primary CB melanoma. Mean patient age was 61.9 ± 12.0 years; 25 (54%) were female, 21 (46%) were male. Metastatic spread occurred on average after 2.9 ± 1.6 years. 45 (98%) developed metastatic spread to the liver, 1 (2%) showed metastatic spread to the lung following primary choroidal or CB melanoma.

**Fig 3:**
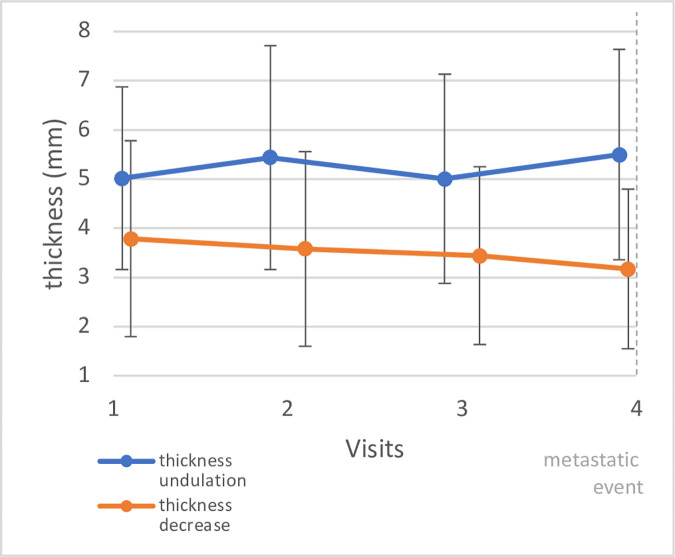
Primary choroidal and CB melanoma thickness are displayed 4 visits before the metastatic event was detected (vertical dashed line). The visits took place every six months. Data are displayed as mean ± 2 SD.

A repeated measures ANOVA of the 46 patients shows that there is a statistically significantly change over time in primary tumor thickness during the four visits before the ME was detected (p = 0.03). Grouping patients based on primary choroidal or CB melanoma thickness change before the ME, shows that 28 patients (61%) show a decrease and 18 patients (39%) presented with an undulating primary tumor thickness profile (see Fig 3).

A repeated measures ANOVA shows a statistically significant change of primary tumor thickness over time in group 1 (undulation, p = 0.019, n = 18) and group 2 (decrease, p = 0.013, n = 28) before a metastatic event occurs (see Fig 3).

Comparing primary tumor thickness between groups 1 (undulation) and 2 (decrease) at diagnosis shows that there is a statistically significant difference (group 1: 3.9 ± 1.5; group 2: 5.0 ± 1.5; p = 0.023). However, time until ME is not statistically different between the groups (p = 0.9).

## Discussion

Up to 30% of primary UM patients develop metastases within ten years despite treatment [17], with numbers varying for follow-up periods [16,20,32]. In patients with primary UM, death may occur 1–3 years after treatment [33,34]. Consequently, indicators for treatment success and metastatic spread are vital when it comes to follow-up and prognosis.

In this study 18.5% of patients with primary choroidal or CB melanoma developed metastases on average after 4.8 ± 3.5 years. This is in accordance with previously published data stating that ‘metastasis occurred in 8%, 15%, and 25% at 3, 5, and 10 years, respectively’ [5].

In a mixed model approach we identified two parameters which are associated with time until metastatic spread; age and primary choroidal or CB melanoma thickness. Interestingly, other parameters previously identified to be associated with primary choroidal melanoma metastasis could not be confirmed in this patient collective. Specifically primary tumor volume, which has lately been shown to improve prognostication of metastatic mortality and to be associated with increasing frequency of monosomy 3, gain of chromosome 8q, and epithelioid cytomorphologic features [35,36], could not be confirmed in this approach. Assuming that this discrepancy in results may be attributed to the method of volume calculation cannot be completely ruled out, as this study and the group of Stålhammar et al. [35] use a similar approach based on an ellipsoidal solid model, yet the latter approximate tumor dimensions assuming tumor width to be 85% of the largest basal diameter [35], rather than ultrasonography based tumor width and length as in this study. Additionally, the datasets the studies are based on are not comparable in numbers; approximately 6500 vs. 380 patients in this approach.

Older age at presentation is listed a feature predictive of poor prognosis for choroidal melanoma. [37] Also, in this study the factor age shows up in a mixed model approach aiming at identifying factors associated with time until metastatic spread. However, there is no statistically significant correlation between age at diagnosis and time until ME. Comparing age at choroidal or CB melanoma diagnosis between patients with (60.5 ± 14.6 years) and without metastatic event (61.6 ± 13.1 years) shows no statistically significant difference between groups (p = 0.54). Consequently, age may be considered an indirect effect in the model calculated. There is further no correlation of age at diagnosis with T-staging and there is no correlation between age and primary tumor thickness at the time of diagnosis, for both, patients with and without recorded metastasis. Overall, mean patient age of this cohort is perfectly in line with previous publications stating that the median age is between 59 and 62 in Caucasian cohorts. [37] Further, an increasing mean age has been recorded during the last 50 years (from 59 to 62 years). [37] Consequently, older age has to be considered an informative parameter in patient communication regarding metastatic risk, as no additional correlation between time until ME and age could be identified. Based on the literature, it is assumed that patients over 60 years of age have higher risks of metastatic spread compared to younger patients [4,38].

Thickness is a robust indicator for malignant transformation, which has lately been confirmed in a machine learning model [39]. The relative risk increases by 2.2 with each millimeter increase in tumor thickness [21]. Another study found a 5% increasing risk for a ME with each millimeter increase in thickness [5]. In line with these studies, the present data show that there is a negative and statistically significant correlation between primary tumor thickness and time until metastasis. In other words: the thicker the primary choroidal or CB melanoma, the earlier the metastatic spread (see Fig 2). When grouping patients based on primary tumor thickness change right before the ME is detected, we found that approximately 60% show a decrease in thickness and 40% of patients an undulating thickness profile. When looking at the longitudinal data of these two groups (see Fig 3), it has to be stated, that the undulation detected is rather a stable thickness profile over time, as the thickness difference between time-points is extremely small (see Fig 3). However, the ‘decreasing’ group shows a continuous decrease in primary tumor thickness. Consequently, primary tumor thickness change before diagnosis of metastasis is no indicator for a ME. Further, there is no statistically significant difference between groups when it comes to time until ME (p = 0.9). Hence, the results of this study are in line with previous findings [40] highlighting, that thickness is associated with ME in patients with primary choroidal or CB melanoma. Fig 2 illustrates this association: the thicker the primary choroidal or CB melanoma, the earlier a potential ME.

In future we are considering recharacterizing patients and including histomorphology and cytogenetic information into the analysis, which at the current state can also be considered a limitation of this study.

In conclusion, this study expands the current knowledge on factors associated with metastasis due to primary choroidal or CB melanoma. So far primary choroidal or CB melanoma thickness has been shown to be a risk indicator for MEs. Now, we could show that primary choroidal or CB melanoma thickness is also correlated with time until ME. The association can be expressed as: the thicker the primary tumor, the earlier the potential ME. In future, we are planning to repeat this analysis once data on tumor histology and genetic alterations become available.
